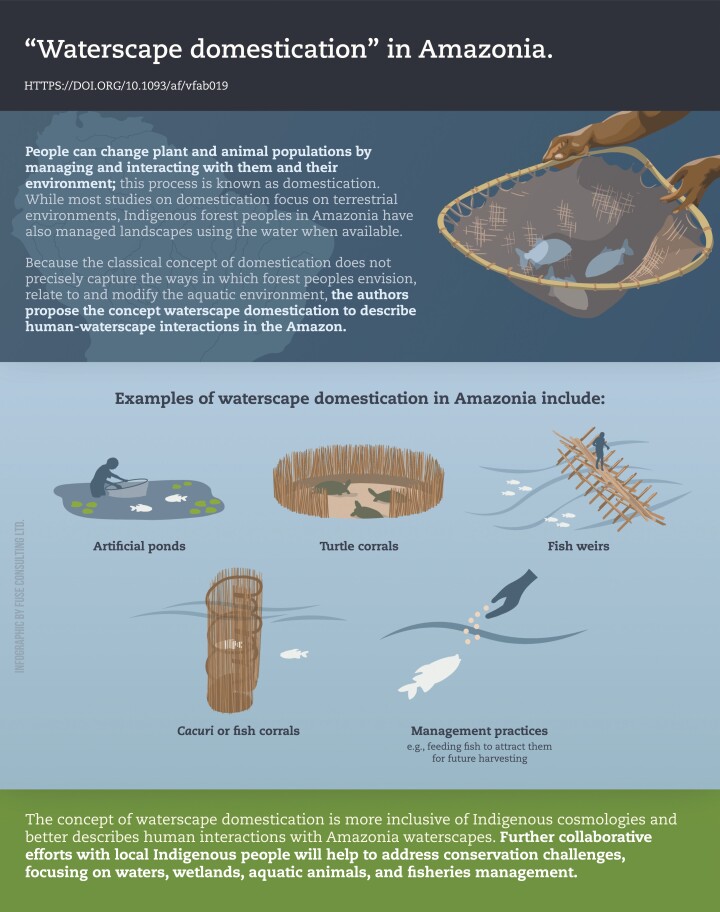# "Waterscape domestication" in Amazonia.

**DOI:** 10.1093/af/vfab029

**Published:** 2021-06-19

Animal domestication was a pivotal point in human history and coincided with a major change in human evolution. The domestication of crops was quickly followed by the domestication of livestock (Cucchi and Arbuckle, 2021). While a major focus of this Animal Frontiers issue is on the origins of domestication, current adaptations to domestication to meet challenges of environmental sustainability (Prestes-Carneiro et al., 2021) and to feeding human populations (Lecoq and Tomey, 2021) are also explored.